# Factors Associated with Types and Intensity of Postoperative Pain following Gynecological Laparoscopic Surgery: A Cross-Sectional Study

**DOI:** 10.1155/2017/2470397

**Published:** 2017-09-13

**Authors:** Chia-Fen Hsien, Chiu-Lin Wang, Cheng-Yu Long, Yung-Hung Chen, Winter Yu-Ning Lee, Shih-Chin Chen, Kun-Ling Lin, Cherng-Jye Jeng, Eing-Mei Tsai, Feng-Hsiang Tang

**Affiliations:** ^1^Department of Obstetrics and Gynecology, Kaohsiung Municipal Siaogang Hospital, Kaohsiung Medical University, Kaohsiung, Taiwan; ^2^Department of Obstetrics and Gynecology, Kaohsiung Medical University Hospital, Kaohsiung Medical University, Kaohsiung, Taiwan; ^3^Graduate Institute of Medicine, College of Medicine, Kaohsiung Medical University, Kaohsiung, Taiwan; ^4^Graduate Institute of Clinical Medicine, College of Medicine, Kaohsiung Medical University, Kaohsiung, Taiwan

## Abstract

**Objective:**

To evaluate influences of various factors on the types and intensity of postoperative pain following gynecologic laparoscopic surgery.

**Study Design:**

Cross-sectional questionnaire and chart review.

**Results:**

A total of 84 questionnaires were distributed and returned. The types of postlaparoscopic pain are different in multiparous women and nulliparous ones (71.43% surgical wound pain versus 63.64% nonsurgical wound pain, *p* = 0.0033) and those with striae gravidarum and without striae gravidarum (93.94% surgical wound pain versus 52.94% nonsurgical wound pain, *p* < 0.0001). On postoperative day 1, the average VAS score is higher in nonsurgical wound pain than in surgical wound pain (5.62 ± 1.50 versus 3.51 ± 1.68, *p* < 0.0001). The CO_2_ removal procedure has a significant negative correlation with the VAS of nonsurgical wound pain (coefficient: −0.4339, *p* = 0.0187).

**Conclusion:**

Our study suggests that women with abdominal rigidity (nulliparous, no striae gravidarum) experience mainly nonsurgical wound pain, while women with abdominal wall laxity mostly experience surgical wound pain. The VAS score of nonsurgical wound pain is greater than surgical wound pain on postoperative day 1. The CO_2_ removal procedure has negative correlation to the VAS score of nonsurgical wound pain on postoperative day 1.

## 1. Introduction

Ever since the beginning of the surgical history, surgeons have continued to make effort pursuing surgical techniques with minimal invasiveness, the intention of which is to minimize trauma and decrease the postoperative pain and shorten the length of hospital stay. The introduction of laparoscopic surgery is revolutionary in this aspect; with the recent advances in the laparoscopic surgical instruments and techniques, laparoscopic surgery can be employed to most surgically indicated patients in the gynecology field [1].

Compared to traditional surgeries, the postoperative discomforts are less in laparoscopic ones. Due to the limited space of the abdominal cavity, pneumoperitoneum is an essential and unique procedure in laparoscopic surgery. However, if the pneumoperitoneum condition is inappropriate, or the CO_2_ removal is not done sufficiently after operation, comorbidities often occur. Furthermore, if CO_2_ builds up below the diaphragm, it will cause irritation of the phrenic nerve, and subsequently results in shoulder pain. Sometimes, this kind of comorbidities may be more debilitating than the incisional wound pain [2–4]. According to the previous studies, the percentage of patients that suffered from postlaparoscopic shoulder pain is in the range of 35%~80%. The severity from mild to severe has all been reported, and the duration of the discomfort may last up to 72 hours after operation [5, 6].

To the best of our knowledge, most of the investigations pertaining to the comorbidities of laparoscopy focus on the surgical complications mainly, such as intestinal trauma, ureter trauma, inferior vena cava compression and injury, and intraoperative CO_2_ temperature/moisture in association to postoperative pain [7, 8]. Studies focused on the factors and the different factors in association with postoperative pain are currently limited in the literature. Thus, we designed this study to observe the factors which would have relationship to the different types and intensity of postlaparoscopic pain.

## 2. Materials and Methods

**Figure 1:**
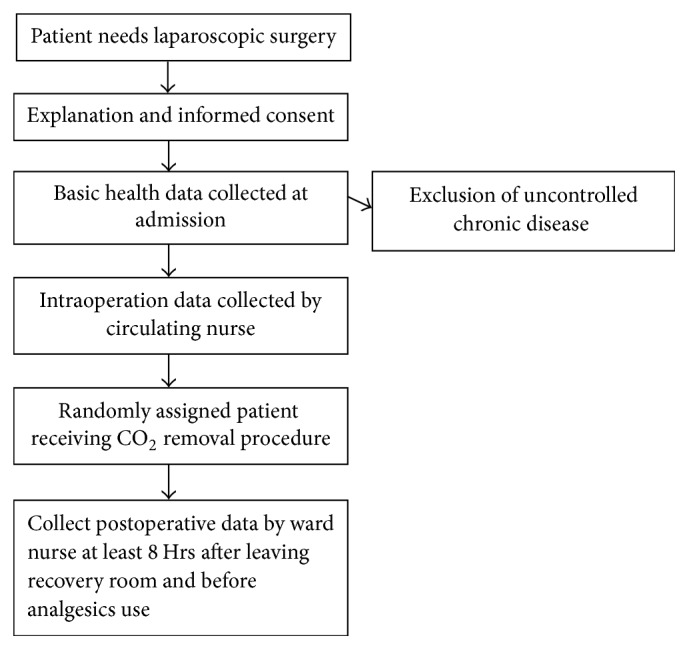
Flowchart of patient selection and enrollment.

This is a cross-sectional study of the relationship between the different factors of laparoscopic gynecologic surgery and the resulting postoperative pain. Subjects who underwent gynecological laparoscopic surgery due to benign gynecologic condition in Kaohsiung Municipal Siaogang Hospital from January 01, 2011, to December 31, 2011, were included. But those who received other surgery at the same time (e.g., tension-free vaginal tape) were excluded from this study. All of the study subjects were capable of communicating effectively with Mandarin and/or Taiwanese, and the study participants' consent was obtained after the subjects were fully informed of the purpose of the study. The flowchart of patient selection and study is shown in Figure 1.

All the laparoscopic surgeries were performed via three trocars insertions, one 10 mm trocar inserted through umbilicus and the other two 5 mm trocars inserted through left lower quadrant of patient's abdomen. After completion of surgery, the 84 patients randomly received CO_2_ removal process by suction of intra-abdominal CO_2_ from 10 mm trocars till the abdominal wall is flat and soft. Other patients who do not receive this procedure would let the intra-abdominal CO_2_ passively flow out from the 10 mm trocar. The two 5 mm trocars were removed before both steps.

The pre- and postmedication of anesthesia are according to the anesthesiologist decision. The analgesic drugs used in perianesthetic period are decided by anesthesiologist. We do not include those who use patient-controlled analgesia machine. We do not use local anesthesia agent before trocar insertion or removal. No special medication was used before insufflation of CO_2_.

In this cross-sectional study, data collection is via chart review and structured nondisguised questionnaires, which included the following:

1. Demographic data: age, parity, height, weight, body surface area, and presence of striae gravidarum or not.
2. Surgical parameters: volume of CO_2_ insufflated, pre-set intra-abdominal pressure, duration of the operation, postoperative CO_2_ removal, volume irrigated during operation, postoperative drainage placement, and laparoscopy procedure performed.
3. Postoperative pain evaluation: includes the types and the intensity of the pain, which is assessed via the visual analogue scale (VAS). The types of postoperative pain are divided into surgical wound pain and nonsurgical wound pain. Nonsurgical wound pain is defined as any postoperative pain not located on the incisional trocar site, such as shoulder, intercostal, epigastric, and subphrenic area.

The demographic data was recorded by ward nursing staff on patient's admission. The presence of striae gravidarum or not and the surgical parameters were recorded by circulating nurse of operating room during operation. The postoperative pain evaluation was recorded by ward nursing staff using questionnaires on the morning of postoperative day 1, and at least 8 hours after patient's returning to ward, but before any forms of analgesics are used after leaving recovery room. A total of 84 questionnaires were distributed and 84 questionnaires were answered and returned. The effective questionnaire return-ratio is thus 100%.

The returned questionnaires were first encoded and then filled with the Excel program. Statistical analysis was performed with the application of the SPSS10.0 software (IBM). Statistical parameters analyzed included the mean, standard deviation, *t* value, Chi-square test, and Pearson correlation.

## 3. Results

A total of 84 subjects who underwent gynecologic laparoscopy surgery were included in this study. Their demographic distributions and the various parameters were shown in Table 1. The mean age of the patient is 40.10 (±11.34) years. The parity condition of those patient is 0 parities in 26.2%, 1~2 parities in 48.8%, and 3 parities or more in 25%. The presence of striae gravidarum on the abdominal skin is observed in 39.3%. The mean BMI is 24.41 kg/M^2^ (±4.87). The mean volume of CO_2_ insufflated during surgery is 204.54 liters (±165.11). Mean setting of intra-abdominal pressure is 14.77 mmHg (±0.7). Mean intra-abdominal irrigation (warmed normal saline) volume during operation is 1272.32 liters (±821.45). Average intraoperative blood loss is 86.79 ml (±139.10). Mean body surface area is 1.65 M^2^ (±0.21). Mean operation time is 1.66 hours (±0.71).

The types of operation received by the 84 patients and the associated demographic data and factors were shown in Table 2. In LAVH group, the age and blood loss amount are significantly more than the adnexa surgery group. In laparoscopic myomectomy (LM) group, the insufflating CO_2_ volume is significantly more than the LAVH group, and the operation time is longer than all other three groups. There are no perioperative complications noted in those 84 patients included. The average VAS scores within different types of operation are not significantly different (*p* = 0.658).

The relationship between various parameters and the types of the postoperative pain are listed in Table 3. According to our study, the nulliparous women experienced mainly nonsurgical wound pain (63.64%), while the multiparous women mostly experienced surgical wound pain (71.43%). The types of pain in the two groups reach significant difference (*p* = 0.0033).

In patients with observed striae gravidarum on her abdominal skin, 93.93% of them experience surgical wound pain. On the contrary, 52.94% of subjects who have no observable striae gravidarum experienced nonsurgical wound pain. The different expression of postoperative pain between the two groups is statistically significant (*p* < 0.0001). Other factors, including intraoperative irrigating volume, intraoperative blood loss, infused CO_2_ volume, patient's BMI, and the use of CO_2_ removal procedure or not, show no significant influences on the types of postoperative pain.

Comparisons of different factors between surgical wound pain group and nonsurgical wound pain group are listed in Table 4. The visual analogue scale (VAS) on postoperative day 1 is statistically significantly different between the two groups (3.51 versus 5.62, *p* < 0.0001). However, in terms of the influences on the duration of the operation or the subject's body surface area in association with surgical or nonsurgical wound pain, neither achieved statistical significance.

The relationship of VAS and the different variables were analyzed via the Pearson correlation and shown in Table 5. The results in surgical wound pain group show that the VAS of the surgical wound pain is inversely correlated with the volume used for irrigation during operation and the intra-abdominal pressure during operation. On the other hand, the VAS of the surgical wound pain is directly correlated with the duration of the operation and the postoperative CO_2_ removal procedure. But neither four factors reach statistical significance.

The VAS of nonsurgical wound pain is directly correlated with intraoperative irrigation volume and intra-abdominal pressure during operation; but operation time is inversely correlated with it. All three factors do not reach significance statistically. Finally, the postoperative CO_2_ removal procedure has significant negative influence on the VAS score of postoperative nonsurgical wound pain.

## 4. Conclusion

Traditionally, postoperative pain is always a big issue for discussion and management. Since the development of laparoscopic surgery, the intensity of postoperative pain had decreased significantly when compared to laparotomy surgery [9]. But due to pneumoperitoneum during laparoscopic surgery, new types of postoperative pain become predominant [10]. There are many researches focused on how to prevent or deal with the nonsurgical wound pain after laparoscopic surgery [11, 12]. But few studies focused on the factors associated with it. In our study, we try to evaluate different factors associated with the two types of postoperative pain, namely, surgical wound pain and nonsurgical wound pain.

The primary result shows that there is a significant difference in the types of postoperative pain in association with the parity. With those who had no childbearing before, the mainly recorded types of postoperative pain are nonsurgical wound pain. With those who had parity before, irrespective of parity number, surgical wound pain is the main type of postoperative pain. Besides, patient with striae gravidarum had a 93.94% ratio whose postoperative pain is surgical wound pain type. When comparing to the result in those women without striae gravidarum, the type of postoperative pain is significantly different. While other factors, including intraoperative irrigating volume, intraoperative blood loss, infused CO_2_ volume, patient's BMI, and the use of CO_2_ removal procedure or not, show no significant influences on the types of postoperative pain. According to our study, it is suggested that the degree of abdominal wall laxity is an important factor to influence the type of postoperative pain. In theory, we believe that nonsurgical wound pain results from distension and irritation to the peritoneum, which result in release of inflammatory substances that stimulate the nociceptors of nerve endings [13]. It is reasonable that in patient with abdominal wall laxity like multiparity or presence of striae gravidarum, there is less stretching and irritation to the peritoneum in the same condition comparing to those whose abdominal wall is more rigid when receiving laparoscopic surgery.

According to our study, patients who suffered from nonsurgical wound pain (e.g., shoulder and epigastric pain) have a higher VAS score compared to those who suffered from surgical wound pain (5.62 versus 3.51, *p* < 0.0001). The need of the pneumoperitoneum procedure with CO_2_ insufflation results in the postoperative hyperstimulation of the diaphragm and the phrenic nerve, the result of which is pain and soreness in nonsurgical sites, such as in the shoulders and epigastric region, causing great discomfort and inconvenience to the patient [14, 15]. But the reason why the degree of nonsurgical wound pain is higher than the surgical wound pain is unknown. Since our study shows that the abdominal wall laxity is an important factor affecting the type of postoperative pain, we believe that those who suffered from nonsurgical wound pain mostly did not experience abdominal wall distension before and have more rigid abdominal wall. So the score of VAS on the postoperative day 1 would be interpreted higher in the nonsurgical wound pain group.

Since the introduction of laparoscopic surgery, there are many researches on the methods to reduce the intensity of nonsurgical wound pain. Generally, reducing CO_2_ retention and decreasing phrenic nerve stimulation by materials are the two main methods. According to the study by Tsai et al. [16], postoperative intra-abdominal injection of 25~30 mL/kg of normal saline solution may facilitate the removal of intra-abdominal CO_2_ gas, resulting in a 40.7% reduction of the ratio of patients suffering from shoulder discomfort and epigastric pain (24 hours after operation) [11, 17]. Phelps et al. [3] also reported that CO_2_ removal by means of pulmonary recruitment maneuver and Trendelenburg position may successfully reduce postlaparoscopic shoulder pain cases to 20%. In our study, we also show that the CO_2_ removal procedure has significantly negative correlation to the VAS score of nonsurgical wound pain on postoperative day 1.

To the best of our knowledge, this study is the first of a few studies on the different factors associated with different types of postoperative pain after laparoscopic gynecologic surgery, which shows that in patient with more rigid abdominal wall, there is more possibility that she will experience nonsurgical wound pain after laparoscopic surgery and that the pain will be more intense. However, CO_2_ removal procedure may have benefit on it. On the other hand, in patient who are multiparous or have striae gravidarum on the abdominal skin, prevention or management of postoperative surgical wound pain may be important. There are limitations to our study, the sample size is not so large, so we cannot observe those factors in just one type of operation, for example, in LAVH. The different operation type may have influence on the result. Besides, VAS score is a subjective indicator, which may be not so precisely described by patient. Further study on it may help us to clarify the mechanism of postoperative pain after laparoscopic gynecologic surgery and develop more effective methods to reduce the intensity of pain in different types in patient receiving gynecologic laparoscopic surgery.

## Figures and Tables

**Table 1:** Demographic distributions and the various parameters.

	Case number (percentage)	Average	Standard deviation	Maxi.	Mean	Mini.
Age (years)	84	40.10	11.34	81	39.5	17
BMI (Kg/M^2^)	84	24.41	4.87	37.96	22.9	16.7
Body surface area	84	1.65	0.21	2.26	1.62	1.3
Parity						
0	22 (26.2%)					
1-2	41 (48.8%)					
≥3	21 (25.0%)					
Striae gravidarum						
No	51 (60.7%)					
Yes	33 (39.3%)					
CO_2_ volume (L)		204.54	165.11	878	154.9	33.7
Abdominal pressure (mmHg)		14.77	0.700	18	15	12
Irrigation volume (mL)		1272.32	821.45	4500	1000	75
Blood loss (mL)		86.79	139.10	1050	50	5
Duration of surgery (hours)		1.66	0.71	4.583	1.583	0.333
Drainage placement						
No	83 (98.8%)					
Yes	1 (1.2%)					

**Table 2:** Demographic data and factors of different types of operation.

Characteristics	LAVH (1) (*N* = 29)	Adnexa (2) (*N* = 24)	Myomectomy (3) (*N* = 12)	Others (4)^*∗*^ (*N* = 19)	*p* value
	Mean ± SD	Mean ± SD	Mean ± SD	Mean ± SD	
Mean age (years)	43.76 ± 6.10	34.63 ± 14.99	39.00 ± 7.73	42.11 ± 12.38	0.022 (a)
BMI	25.90 ± 5.53	23.57 ± 5.21	24.18 ± 3.91	23.44 ± 3.54	0.223
BSA (M^2^)	1.68 ± 0.19	1.63 ± 0.21	1.66 ± 0.19	1.60 ± 0.21	0.493
Parity					
0	0 (0.0%)	12 (50.0%)	2 (16.7%)	8 (42.1%)	0.001
1-2	5 (45.5%)	1 (4.2%)	3 (25.0%)	2 (18.2%)	
≥3	24 (47.1%)	11 (45.8%)	7 (58.3%)	9 (17.6%)	
Previous surgery					
No	18 (62.07%)	14 (58.33%)	10 (83.33%)	11 (57.89%)	0.461
Yes	11 (37.93%)	10 (41.67%)	2 (16.67%)	8 (42.11%)	
Striae gravidarum					
No	14 (48.3%)	17 (70.8%)	8 (66.7%)	12 (63.2%)	0.371
Yes	15 (51.7%)	7 (29.2%)	4 (12.1%)	7 (36.8%)	
CO_2_ volume (ml)	184.19 ± 131.57	128.5 ± 55.48	401.17 ± 253.37	213.61 ± 148.16	<0.001 (b)
Mean intra-abdominal pressure (mmHg)	14.72 ± 0.53	14.92 ± 0.93	14.67 ± 0.49	14.72 ± 0.75	0.695
Irrigation volume (ml)	1316.38 ± 825.55	1358.33 ± 686.46	866.67 ± 619.87	1377.78 ± 1065.81	0.331
Intraoperative blood loss Volume (ml)	161.55 ± 202.06	25.42 ± 30.29	76.25 ± 86.74	54.44 ± 71.12	0.002 (a)
Duration of surgery (hours)	1.84 ± 0.49	1.12 ± 0.38	2.42 ± 0.84	1.52 ± 0.62	<0.001 (c)
Average VAS score	1.30 ± 0.12	1.11 ± 0.14	1.08 ± 0.19	1.13 ± 0.15	0.658

^*∗*^Patients who received adhesiolysis at the same time were included in this group; (a) (1) > (2); (b) (3) > (1); (c) (3) > (1), (3), (4).

**Table 3:** Types of the postoperative pain.

	Types of the experienced pain		*p* value
	Surgical wound pain	Nonsurgical wound pain^*∗*^	
	*N* (%)	*N* (%)	
Parity			0.0033
0	8 (36.36)	14 (63.64)	
1-2	32 (78.05)	9 (21.95)	
≥3	15 (71.43)	6 (28.57)	
Striae gravidarum			<0.0001
No	24 (47.06)	27 (52.94)	
Yes	31 (93.94)	2 (6.06)	
Intraoperative irrigation volume (mL)			0.3040
≤1000	38 (74.51)	13 (25.49)	
>1000	17 (51.52)	16 (48.48)	
Blood loss (mL)			0.4172
<100	35 (62.50)	21 (37.50)	
≥100	20 (71.43)	8 (28.57)	
CO_2_ volume (L)			0.1987
≤200	32 (60.38)	21 (39.62)	
>200	23 (74.19)	8 (25.81)	
BMI (Kg/M^2^)			0.9303
≤24	29 (65.91)	15 (34.09)	
>24	26 (65.00)	14 (35.00)	
Postoperative CO_2_ removal procedure			0.2395
Yes	49 (63.64)	28 (36.36)	
No	6 (85.71)	1 (14.29)	

^*∗*^Nonsurgic wound pain includes shoulder pain, intercostal pain, and epigastric pain.

**Table 4:** Comparisons of different types of postoperative pain.

	Case number (%)	Mean ± SD	*p* value
Postoperative day 1 (VAS)			<0.0001
Surgical wound	55 (65.48)	3.51 ± 1.68	
Nonsurgical wound	29 (34.52)	5.62 ± 1.50	
Duration of the operation (hr.)			0.8982
Surgical wound	55 (65.48)	1.65 ± 0.74	
Nonsurgical wound	29 (34.52)	1.67 ± 0.65	
BSA			0.6178
Surgical wound	55 (65.48)	1.64 ± 0.20	
Nonsurgical wound	29 (34.52)	1.67 ± 0.23	

**Table:** (a) Surgical wound pain

Variable 1	Variable 2	Correlation coefficient	*p* value
Intraoperative irrigation volume	VAS	−0.0656	0.6343
Intra-abdominal pressure	VAS	−0.2443	0.0722
CO_2_ removal procedure	VAS	0.2046	0.1341
Operation time	VAS	0.0949	0.4909

**Table:** (b) Nonsurgical wound pain

Variable 1	Variable 2	Correlation coefficient	*p* value
Intraoperative irrigation volume	VAS	0.1588	0.4105
Intra-abdominal pressure	VAS	0.1262	0.5143
CO_2_ removal	VAS	−0.4339	0.0187
Operation time	VAS	−0.0618	0.7502
